# Glycemic Status Assessment by the Latest Glucose Monitoring Technologies

**DOI:** 10.3390/ijms21218243

**Published:** 2020-11-03

**Authors:** Ilaria Malandrucco, Benedetta Russo, Fabiana Picconi, Marika Menduni, Simona Frontoni

**Affiliations:** 1 Unit of Endocrinology, Diabetes and Metabolism, S. Giovanni Calibita, Fatebenefratelli Hospital, 00186 Rome, Italy; ilariamalandrucco@outlook.com (I.M.); benedetta_russo6@msn.com (B.R.); fabipicco@gmail.com (F.P.); 2 Department of Systems Medicine, University of Rome Tor Vergata, 00133 Rome, Italy; marika.menduni@gmail.com

**Keywords:** continuous glucose monitoring (CGM), ambulatory glucose profile (AGP), integrated glycemic state (IGS)

## Abstract

The advanced and performing technologies of glucose monitoring systems provide a large amount of glucose data that needs to be properly read and interpreted by the diabetology team in order to make therapeutic decisions as close as possible to the patient’s metabolic needs. For this purpose, new parameters have been developed, to allow a more integrated reading and interpretation of data by clinical professionals. The new challenge for the diabetes community consists of promoting an integrated and homogeneous reading, as well as interpretation of glucose monitoring data also by the patient himself. The purpose of this review is to offer an overview of the glycemic status assessment, opened by the current data management provided by latest glucose monitoring technologies. Furthermore, the applicability and personalization of the different glycemic monitoring devices used in specific insulin-treated diabetes mellitus patient populations will be evaluated.

## 1. Introduction

Diabetes mellitus (DM) is a chronic metabolic disease with a rapidly growing prevalence. According to the International Diabetes Federation (IDF), a total of 463 million people aged 20–79 years are estimated to be living with diabetes, representing 9.3% of the global adult population. Type 1 diabetes mellitus (T1D) accounts for 5 to 10% of DM and type 2 diabetes mellitus (T2D) accounts for around 90% of all cases of diabetes [1]. Controlling glycemia in both T1D and T2D remains key to optimize the effectiveness of treatment, to reduce the risk of hypoglycemia, and prevent microvascular complications and reduce long-term risk of macrovascular diseases [2].

Structured self-monitoring of blood glucose and continuous glucose monitoring are valuable tools to improve glycemic control [3]. Several glucose monitoring systems are currently available for daily diabetes self-management ranging from self-monitoring of blood glucose (SMBG) to continuous glucose monitoring (CGM) up to the most emerging devices. Each has its own technology with specific strengths and limitations that can impact their usefulness and acceptability within specific patient populations. Furthermore, integrated insulin pump and glucose monitoring systems are now increasingly available [4].

ADA recommends to personalize glucose monitoring in each DM patient, according to the patient’s characteristics, clinical needs, lifestyle, and treatment, by an accurate assessment of the different device technologies as well as integrated systems [4].

As far as advanced and performing technologies of glucose monitoring systems become available, the diabetes healthcare team faces an increasing number of available glucose data, that need to be properly read and interpreted, in order to get the best benefit for the patient. This is time-consuming and requires an adequate formation of the diabetes team, to make therapeutic decisions personalized on the patient ’s metabolic needs, as much as possible. For this purpose, new parameters have been developed, to allow a more integrated reading and interpretation of data by clinical professionals and consequent homogeneous therapeutic decisions, to be shared by the whole scientific community. The use of time in range (TIR), glucose management indicator (GMI), and ambulatory glucose profile (AGP) are currently recommended [5]. However, CGM system not only provides a method of collecting and using glucose data for the diabetology team “perspective”, but also for the patient “perspective”. The new challenge for the diabetes community therefore consists in promoting an integrated and homogeneous reading, as well as interpretation of glucose monitoring data also by the patient himself. The final goal is to educate him/her to make therapeutic decisions as close as possible to his/her metabolic needs.

The purpose of this review is to offer an overview of the glycemic status assessment, opened by the current data management provided by latest glucose monitoring technologies. Furthermore, the applicability and personalization of the different glycemic monitoring devices used in specific insulin-treated DM patient populations will be evaluated.

## 2. Data Management

Hemoglobin A1c (HbA1c) is currently recognized as the key marker for assessing the risk of long-term diabetes complications in T1D and T2D patients. However, HbA1c used alone may be insufficient to optimally guide a personalized therapy change as it is does not provide information about acute glycemic excursions and cannot reveal duration or timing of hypo and hyperglycemia, or the presence of glycemic variability [6].

The relationship between glycemic variability (GV) and HbA1c remains under debate [7]. Although data from the literature are still controversial, GV is likely to be incompletely expressed by HbA1c, particularly in patients with good metabolic control [8]. Although there is a positive correlation between GV and the mean glucose, GV itself affects HbA1c to a very small extent due to the very slow pace of haemoglobin glycation process [9].

The relationship between GV and diabetic complications has gained much interest and has been evaluated. Evidence reported that some GV indexes are associated with diabetic retinopathy [10], retinal neurodegeneration [11], autonomic and peripheral neuropathy [12,13], and endothelial and cardiovascular damage in T2D patients [14]. Hyperglycemic peak has been associated with a reduction in cerebral vascular reactivity, even before the onset of overt diabetes [15].

### 2.1. Data Management: Diabetology Team “Perspective”

The use of CGM allows for the direct observation of glycemic excursions and daily glucose profiles, which can inform on immediate therapy decisions and/or lifestyle modifications [5]. CGM provides sufficient data to generate a representative CGM-derived mean glucose value for a given patient. From this mean glucose, and using a standard formula, an estimated HbA1C can be generated, intended to approximate the value of a simultaneously measured laboratory HbA1C. Recently a new term was selected to replace estimated HbA1c, named glucose management indicator (GMI) generating a new formula for converting CGM-derived mean glucose to GMI. The GMI with CGM metrics provides information for a more personalized diabetes management [6].

The CGM-derived parameters include the time in range (TIR), which represents a glycemic target between 70 and 180 mg/dl for T1D and T2D patients, the time above range (TAR) (>180 mg/dl) and the time below range (TBR) (<70 mg/dl). The TIR can be personalized according to the patient’s characteristics, in order to achieve a glycemic control compliant to the specific needs of the single patient. Establishing target percentages of time in the various glycemic ranges with the ability to adjust the percentage cut points to address the specific needs of special diabetes populations (pregnancy, high-risk) facilitates safe and effective therapeutic decision making within the parameters of the established glycemic goals [5].

The relationship of HbA1c to TIR in patients with diabetes has been widely demonstrated [16]. In addition, associations of TIR with the development or progression of diabetic retinopathy and microalbuminuria was demonstrated in T1D patients [17]. A similar association between TIR and diabetic retinopathy was observed in a recent study of CGM metrics in T2D patients [18].

The availability of data for the evaluation of glucose profile is of fundamental importance for an accurate and significant interpretation of CGM. Ambulatory glucose profile (AGP) is an analytical method that allows easy and visual understanding of glucose data. The AGP report incorporates all the core CGM metrics and targets along with a 14-day composite glucose profile as an integral component of clinical decision making [19].

The diabetes community is promoting the integration of information on the diabetology team “perspective” in order to obtain composite metrics, potentially able to provide a more complete and clinically useful picture about glycemic control with respect to traditional individual metrics. Using composite metrics derived from CGM, a new multicomponent composite metric has been developed: the comprehensive glucose pentagon (CGP), which depicts glycemic control both numerically and visually. The CGP includes several parameters that have not been previously considered in composite metrics of glycemic control: mean sensor glucose, glucose variability, severity of hypo- and hyperglycemia, and time out of range, i.e. the reciprocal of time in range, while eliminating A1C as an integral component. This new multicomponent composite metric has the potential to enable health care providers and patients to better understand the different components of glycemic control and the effect of various interventions on the individual elements [20].

In order to make glucose information collected available to healthcare professionals, patients, and caregivers, many platforms enable patients to share their personal glucose profiles and data with caregivers and healthcare professionals. On one side, this allows more frequent insulin dosing adjustments and faster clinical interventions [21]. On the other side, however, the wide amount of glucose related data may complicate the insulin dosing decision-making for patients as well as for health care professionals. Digital tools and decision support systems may guide insulin adjustment in certain situations such as eating and physical activity in order to help patients on therapeutic decisions [22,23,24]. Recently, decision support systems based on algorithms have been developed to help clinical professionals to make an accurate therapeutic intervention [25].

### 2.2. Patient “Perspective”: Data Management: Integrated Glycemic State

The transition from self-monitoring of capillary blood glucose to continuous glucose monitoring changes the paradigm of home glucose monitoring also on the patient “perspective”. Self-monitoring of capillary blood glucose provides a finger-prick method of collecting and using the glucose data describing a point “blood glucose value” [26]. On the other hand, CGM provides a multiparametric method for collecting and using glucose data, which consists of three parameters simultaneously available to the patient at all times: (1) the point value of blood glucose; (2) the trend arrow that indicates the direction and speed towards which blood glucose is moving; (3) glucose profile of the previous hours/days. Compared to the finger-prick value of blood glucose, these parameters allow for a more comprehensive interpretation of the patient’s glucose condition, allowing a more adequate therapeutic decision [27].

Following the above, we defined this multiparametric model of glucose profile as integrated glycemic state (IGS). The IGS indicates the glycemic status in the single patient, at a specific moment, derived from a multiparametric evaluation that includes the three CGM parameters previously described.

The IGS moves from the concept of a “glycemic value” to a “glycemic state” on the patient side. This allows a new awareness by the patient, that will be able to make therapeutic decisions based not on a single glycemic value but on a glycemic state, determined by the integration of the three CGM parameters. He, therefore, needs to be educated in the interpretation of this glycemic state; since for the same blood glucose value, a different therapeutic action is required, depending on whether he/she is in a condition of a rapidly descending or rapidly rising trend arrow or on a previous hypo- or hyperglycemia.

Therefore, the IGS includes numerous information that describes the patient’s glycemic condition at that moment in a more real way, however, it requires a greater ability to interpret the data by the patient in order to reach an appropriate therapeutic decision to the metabolic needs of that moment. In this perspective, the IGS contributes to the realization of precision medicine.

As in the interpretation of the glycemic control data by the diabetology team, where AGP does not replace HbA1c but integrates and completes it [5], in the interpretation of the glycemic control data by the patient we move from the “glycemic value” to the “glycemic state” where, here too, the latter does not replace the former but integrates and completes it.

The IGS, which is an intrinsic data of the patient, fits into the environment in which the patient is at that precise moment. The environment may include meals, physical activity, emotional stress, health, or illness [28].

**Figure 1:**
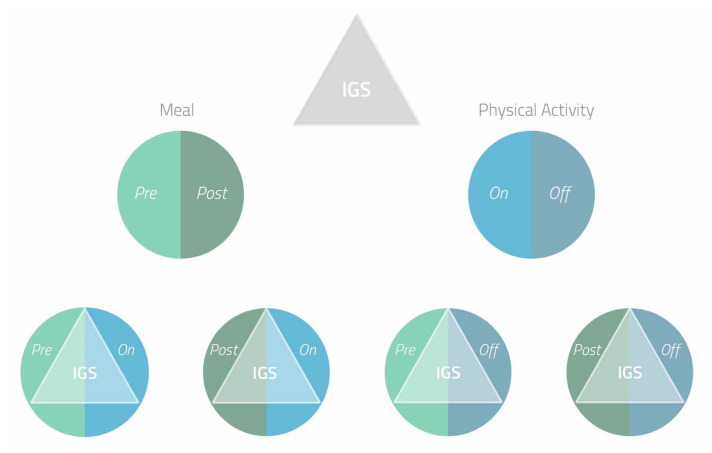
Integrated glycemic state (IGS) in four different conditions. IGS includes the point value of blood glucose, the trend arrow that indicates the direction and speed towards which blood glucose is moving, glucose profile of the previous hours/days. The same IGS can occur in four different conditions: pre-meal in the presence of physical activity, pre-meal in the absence of physical activity, post-meal in the presence of physical activity, post-meal in the absence of physical activity. For each condition the patient will be able to make different therapeutic decisions based on IGS.

Among these the most significant are meal and physical activity. Therefore, the same IGS can occur in four different conditions: pre-meal in the presence of physical activity, pre-meal in the absence of physical activity, post-meal in the presence of physical activity, post-meal in the absence of physical activity. For each of these conditions, the patient will be able to make different therapeutic decisions based on IGS (Figure 1).

### 2.3. Data Management: “Circular Flow”

As previously discussed, the data produced by glucose monitoring systems are available to the diabetes healthcare team and patients, and a correct interpretation of the data is essential to allow an appropriate therapeutic intervention [5].

**Figure 2:**
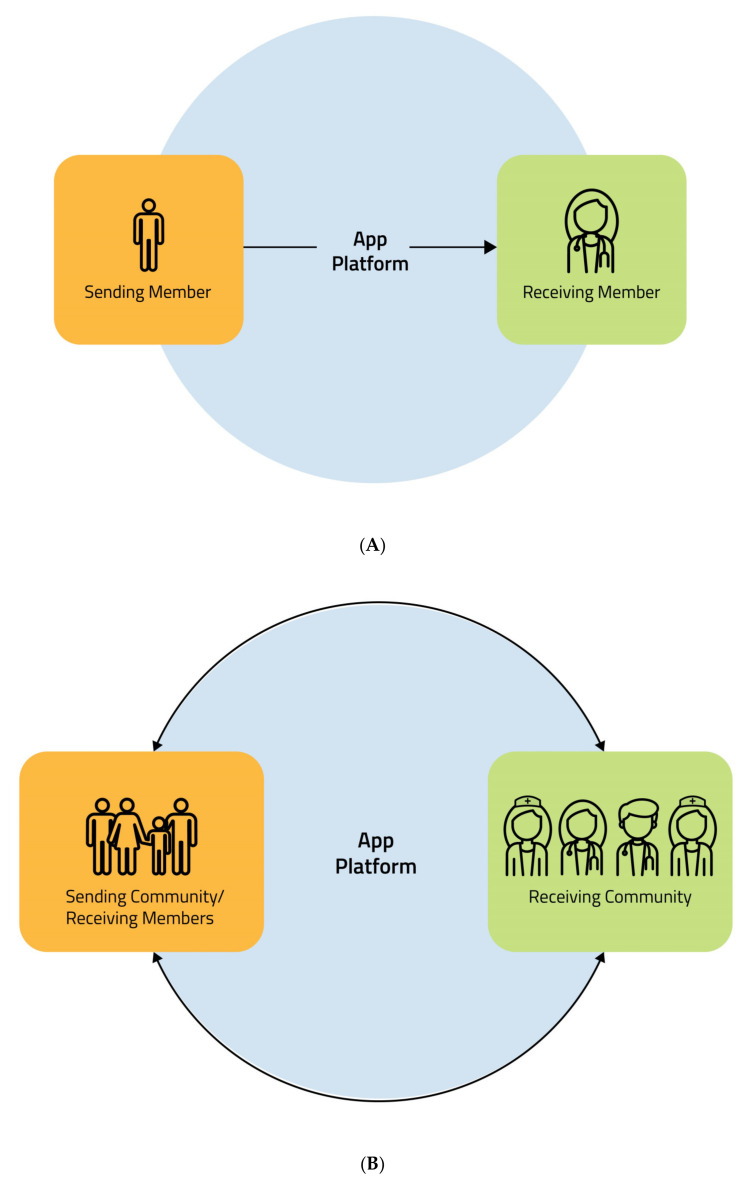
(**A**) Data sharing. The patient, identified as “sending member”, shares its own glucose profile data with the healthcare professionals, identified as the “receiving member”. (**B**) Data sharing: circular flow. The patient supported on the management of the diabetes by family members, cohabitants and caregivers, that we identify as “sending community”, shares glucose profile data with the multidisciplinary diabetes team, that we identify as “receiving community”, according to a model that we propose to call “circular flow”. The members of the sending community can be themselves receiving members.

Through the platforms dedicated to data management, the patient, identified as “sending member”, shares its own glucose profile data with the healthcare professionals, identified as the “receiving member” (Figure 2A).

In routine clinical practice the patient in most cases is not alone in the management of diabetes and glycemic data, but is supported by family members, cohabitants, and caregivers. Similarly, the diabetologist is not the only one to have access to patient glucose data but is part of a multidisciplinary team in which several professional figures collaborate with respective roles and have access to the management of glycemic data sent by patients [29]. Therefore, in the management of glycemic monitoring data it is probably more correct to use the terms “sending community” and “receiving community” with an exchange of data according to a model that we propose to call “circular flow”. Moreover, the members of the sending community can be themselves receiving members (Figure 2B). The more people involved in this process, the more it is necessary to have a unique language in order to avoid a distorted interpretation of the glycemic data that can lead to suboptimal therapeutic decisions. Therefore, this process also requires adequate education and technology itself comes to our aid. In fact, as was experienced during the COVID-19 emergency, virtual training platforms represent tools for alternative educational paths that can be complementary to traditional ones. Virtual training can be accessed by multiple people at the same time even if they are in different places and this greatly facilitates the educational process [24].

## 3. Current Glucose Monitoring Systems

Self-monitoring of blood glucose (SMBG), based on capillary glucose testing, remains the longest-used method to monitor glucose levels and to maintain glycemic control in insulin treated DM patients [30].

SMBG devices are ultraportable with a good accuracy and sensitivity but only provide a snapshot of the glucose level at one specific point in time. The requirement to perform a fingerstick to obtain a blood sample can be time consuming, inconvenient, and painful, consequently leading to poor compliance thus limiting the potential benefits of SMBG [26]. Moreover, long-term trends in glucose fluctuations as a result of insulin therapy, diet, and lifestyle choices are not accurately reflected [31].

SMBG technology is constantly evolving and may have different advanced technologies up to SMBG device with automated bolus calculator with the goal to suggest patients the dose of insulin for the meals; there is evidence of an improvement in self-decision and in glycemic control, in patients using SMBG with this technology [32,33]. Several data shows that the automatic bolus calculator built into the glucometer is effective in improving blood sugar control and preventing hypoglycemic episode [34]. In addition, the evolution of mobile technology led to development of smartphone applications related to the use of image processing technology, e.g., GoCARB system, and voice recognition technology, e.g., VoiceDiab system, that recognize meals and provide decision support for prandial insulin dosage [35].

Over the past few years continuous glucose monitoring (CGM) has changed the way to monitor blood glucose. This technology provides a continuous measurement of glucose concentrations in the interstitial fluid as a way to optimize glucose control by keeping levels in their target in range for a longer period of time, thereby improving HbA1c. CGM systems consist of a disposable sensor that measures glucose concentration and can be inserted or implanted subcutaneously, and an external transmitter that stores glucose values and sends the values to a receiver as standalone or integrated on insulin pump or smartphone. The sensor is functioning from a minimum of 7–14 days for the inserted subcutaneous sensor to a maximum of 180 days for the implanted subcutaneous sensor [36]. The glucose data are either manually scanned or wirelessly transmitted to a nearby receiver to display the readings. Patients and their diabetology team are able to assess the glucose profile, which helps them to make appropriate decisions about therapy, diet, and physical activity [31].

Three types of CGM systems are now available: realtime CGM (rt-CGM), retrospective CGM (r-CGM), and intermittently scanned CGM (is-CGM) or flash glucose monitoring (FGM). The rt-CGM systems measure glucose levels continuously and provide automated alarms and alerts at specific glucose levels. Therefore, it is aimed at well-trained patients, capable of making decisions on changing insulin rate, according to recorded glucose levels. On the contrary, glucose levels measured by r-CGM systems are not displayed to the patient in real time, and they can be analyzed in a retrospective manner, by the physician. The Is-CGM or FGM systems measure glucose levels continuously but only display glucose values when the patient scans the sensor [4,37]. The latest FGM device presents alarms for hypoglycemia and hyperglycemia [38].

Some models of CGM require once or twice daily fingersticks with SMBG for calibration, while others can be used without calibration [39].

Whether or not to use the CGM data to make therapeutic decisions depends on the reliability of the device and its MARD (mean absolute relative difference). MARD is used to assess the accuracy of CGM devices. Some of the first commercially available CGMs had a published MARD of >20%. The latest generation of CGM devices has a MARD < 10% [40]. Numerous randomized controlled trials (RCTs) have demonstrated improved glucose control in terms of reduced HbA1c in individuals using CGM compared to those using SMBG [39].

## 4. Glucose Monitoring in Different Populations

### 4.1. T1D Children, Adolescents, and Youth Adults

In the analysis of data from almost 27,000 children and adolescents with T1D, treated with multiple day injection (MDI) or in continuous subcutaneous insulin infusion (CSII), an increased daily frequency of SMBG was associated with better metabolic control [41]. This study also demonstrated a decreasing rate of SMBG when age increased: an average of 6.0 measurements/day was found in children aged < 6 years, vs. 5.3 measurements/day in children aged 6–12 year and 4.4 measurements/day in those aged > 12 years. Moreover, a higher frequency of SMBG in CSII group (5.3 measurements/day) compared to MDI group (4.7 measurements/day) was reported.

According to a recent study, advanced technologies of SMBG increased compliance with glucose monitoring in the population of T1D adolescents and poorly controlled young adults [42] suggesting that innovative technology devices encourage treatment adherence in this group of patients [43].

Within children and adolescent populations, a special consideration should be given to the hypoglycemia, particularly in young children (aged < 6 years) who are often unable to recognize, articulate, and/or manage hypoglycemia. A multicenter clinical trial evaluated the effect of rt-CGM on hypoglycemia, in relatively well-controlled children and adolescents using CSII or MDI, and demonstrated a significant reduction by half in time spent in hypoglycemia and a concomitant decrease in HbA1c in this population [44]. Moreover, the SWITCH study showed that the combination of CGM with CSII resulted in significant reductions in mean HbA1c levels and in the proportion of time with hypoglycemia in pediatric patients [45]. However, data from RCT reported that CGM did not improve glycemic control in children aged 4–9 years using insulin pump or MDI despite a high degree of parental satisfaction with CGM, possibly due to a limited use of the CGM glucose data in day-to-day management [46].

Although the use of insulin pump and CGM as standalone devices was demonstrated to be beneficial for glycemic control, the best performance has been obtained by connecting the two devices in an integrated system. Sensor-augmented insulin pump therapy (SAP), combining CSII and CGM, was shown to significantly decrease HbA_1c_ without an increase in hypoglycemia compared with MDI, in a large multicenter trial in children [47]. The availability of insulin pumps communicating with CGM sensors has stimulated the development of algorithms for the automatic suspension or attenuation of basal insulin delivery when hypoglycemia is either detected or predicted from CGM readings [48]. A recent study reported a significant reduction of hypoglycemic events in children and adolescents in SAP with predictive low glucose suspend (PLGS) compared to patients with insulin pump and CGM as standalone devices [49].

More innovative algorithms have made available a hybrid closed-loop system that includes CGM and CSII without patient intervention to adjust basal insulin, which automatically reduces, increases, and suspends basal insulin delivery [21]. A recent study suggested that use of day-and-night hybrid closed-loop (HCL) in suboptimally controlled adolescents with T1D is safe, feasible, and increases the time in range [50]. A later study that assessed the effectiveness of the in-home use of a HCL in adolescents with T1D reported an increased time in range, and reductions in HbA1c, hypoglycemia, and hyperglycemia [51]. Moreover, a study conducted subsequently on young people with T1D reported that HCL insulin delivery was safe both during and after physical activity maintaining glucose values mostly within the target range without an increased risk of hypoglycemia [52].

Among the innovative DM management strategies, telemedicine i.e., the use of medical information exchanged from one site to another via electronic communications, is recognized as one of the most relevant for young people with T1D [53]. Telemedicine includes a growing variety of applications and services using two-way video, e-mail, smart phones, wireless tools, and other forms of telecommunications technology. Specialty diabetes care delivered via telemedicine was shown to be safe and has been associated with time savings, cost savings, high appointment adherence rates, and high patient satisfaction [54].

In particular, telemedicine has emerged as a tool that can optimize the management of T1D patients living in rural communities. A study conducted on rural pediatric patients with T1D showed that telemedicine was not inferior to outreach clinics and face-to-face care in controlling HbA1c. Moreover, it demonstrated that patients and caregivers spent less time away from school and work to attend visits and that adherence to appointments was higher [55].

In addition, with the availability of internet and smart phone applications, it has been demonstrated that this technology improves glucose monitoring adherence with high level of satisfaction in T1D adolescent patients [56,57,58].

### 4.2. T1D and T2D Insulin-Treated Adults

SMBG is considered an integral part of treatment for people with T1D and T2D insulin-treated [4]. SMBG usage between 1993 and 2009 increased from 67 to 90% in insulin-treated T2D patients [59]. T1D and T2D insulin-treated patients present the same goals for performing SMBG [30] and it has been demonstrated that more frequent measurements of SMBG were associated with better metabolic control in both T1D and T2D insulin-treated patients [60]. The Multicenter SPA-EDU study showed that the use of structured SMBG combined with intensive education was associated with clinically significant reductions in HbA1c, increased SMBG frequency, and improved quality of life in T1D and T2D insulin-treated patients [61]. Moreover, a recent study conducted only in T2D insulin-treated patients demonstrated that the education of family members in diabetes self-glucose monitoring reduces the diabetes distress and improves autonomy and self-confidence [62].

In the last few years the effectiveness of CGM in T1D and T2D insulin-treated was widely studied; it has been demonstrated that both T1D and T2D insulin-treated patients in MDI who used CGM improve glycemic control and quality of life compared to those using SMBG [63,64,65]. Moreover, a study conducted in T1D patients reported that the greater the time of use of CGM the better the glycemic control [66].

One of the most important applications of CGM is for the management of patients with frequent severe hypoglycemia, often associated with hypoglycemia unawareness. A study conducted on T1D patients showed a reduction of time spent in hypoglycemia associated with the use of CGM compared to SMBG [44]. However, a multicenter randomized control-trial comparing insulin-pump with MDI and CGM vs. SMBG reported that the hypoglycemia awareness can be improved and fear of hypoglycemia can be reduced in both T1D with MDI and SMBG and T1D with CGM and CSII when it is provided a structured education and a continuous support for patients, although satisfaction was higher with CGM and CSII [67]. This study highlights the importance of the structured approach, patient education, and follow-up in specialized centers as the determining factor in reducing hypoglycemia and improving the awareness of hypoglycemia, even independently of the technology.

Studies have been conducted evaluating the efficacy of FGM on glycemic control compared to SMBG in patients with TD1 and T2D insulin-treated with MDI or CSII. In both studies the results report a significant reduction in the time spent in hypoglycemia, in particular during the night, in patients with FGM while no differences are observed in HbA1c change [68,69]. However, a recent study conducted on T2D patients with MDI showed that FGM may lead to amelioration of glycemic control and improved treatment satisfaction [70]. In addition, a recently published UK real-world study, that involved a wide sample of T1D patients, showed that the use of FGM was associated with significantly improved glycemic control and hypoglycemia awareness and a reduction in hospital admissions [71].

The combination of a CGM device with CSII (SAP) in T1D patients has been shown to have greater efficacy in reducing HbA1c compared with MDI. These results were achieved without increasing the frequency or severity of hypoglycemic events and the SAP was associated with improvements in treatment satisfaction and diabetes related distress [72].

A more recent study conducted in patients with T1D and hypoglycemia unawareness reported that the use of SAP with low glucose suspend (LGS) function significantly reduced the incidence of severe hypoglycemia, compared to standard insulin pump therapy [73]. In addition, it has been recently demonstrated that the use of SAP with PLGS reduces hypoglycemia events and duration in T1D patients [74].

Clinical studies have shown a consistent increase in the percentage of time in range, a modest reduction in the percentage of time above range, and reduction in the percentage of time below range in T1D adults who used a hybrid closed-loop system compared with those who used SAP [75,76]. Improvement during the nocturnal period is consistently superior to that observed for the full 24-h period [77]. In addition, a multicenter study conducted on T1D adults has demonstrated that overnight closed-loop system in a supervised outpatient setting improves glucose control with less hypoglycemia overnight and extended those benefits to glucose control during the day [78].

In a study that investigated the safety of a hybrid closed-loop system in patients with T1D, hybrid closed-loop automated insulin delivery was associated with few serious or device-related adverse events [79]. Moreover, it has been reported that the hybrid system delivery provides an effective means to reduce the risk of nocturnal hypoglycemia while increasing the percentage of time spent in target range after afternoon exercise in T1D [80].

Dual-hormone artificial pancreas that deliver glucagon as well as insulin have also shown benefits; Russell et al., showed how use of a dual-hormonal artificial pancreas (AP) could improve time spent in euglycemia and reduce time spent in hypoglycemia and hyperglycemia [81].

Although the use of new technologies relating to monitoring systems with integrated systems may be encouraging for some patients, for others it may have limitations especially for elderly people due to the ability of the patient required to understand and use the system and the necessity for active involvement [82]. However, a study conducted in middle aged and older T2D insulin-treated patients has demonstrated that attitude and behavior related to new glucose monitoring technologies could change over time [83]. In addition, it has been shown that tele-health regarding virtual health coaching related to the several glucose monitoring technologies improves adherence to the use of glucose monitoring devices and diabetes self-management in T2D insulin-treated patients, with consequent improvement of glycemic control [84].

### 4.3. Pregnancy Insulin-Treated

Glucose monitoring during pregnancy is indispensable for improving glycemic control and reducing the risk of maternal and fetal complications [85].

A RCT that investigated the effect of SMBG and CGM on maternal and neonatal complications in women with insulin-treated gestational diabetes reported no significant differences in improvement of HbA1c and in prenatal or obstetric outcomes between the CGM and SMBG groups. However, a lower weight gain was observed in women who used CGM [86].

A recent study conducted on pregnant women with T1D has observed a greater drop in HbA1c, an increased time in range, and a reduction in some adverse neonatal outcomes in women using CGM. Importantly, the improvements in glucose control were equal for women using MDI and women using insulin pumps [87]. In contrast, Secher et al., did not find evidence of an improvement in glycemic control or pregnancy outcomes in their study of 123 women with T1D in MDI or insulin pump that used intermittent CGM [88]. The same authors evaluated self-reported satisfaction and barriers to initiating real-time continuous glucose monitoring in early pregnancy women with T1D and the results demonstrated that the majority of pregnant women with diabetes found real-time continuous glucose monitoring useful. Nevertheless, continuous glucose monitoring was frequently removed earlier than planned, primarily because of skin irritation, technical problems, and inaccuracy [89].

In the last few years emerging technologies have been tested even in pregnant women with diabetes. A recent study conducted on pregnant women with T1D has assessed the safety, efficacy, and longer-term feasibility of day-and-night closed-loop insulin delivery versus SAP therapy. This study demonstrated that closed-loop insulin delivery was associated with comparable glucose control and significantly less hypoglycemia than SAP therapy [90].

Farrington et al., explored the experiences of pregnant women with T1D using closed-loop insulin delivery. The pregnant women with T1D reported experiencing a feeling of improved glycemic control, excitement toward the technology, and empowerment regarding their diabetes. However, they also noted concerns about the cumbersome nature of the devices, problems with technical glitches and alarms [91].

## 5. Cost-Effectiveness

Currently, resources are often limited, and their correct allocation is an important element for the proper functioning of health systems, both scientific communities and payers pay close attention to the cost-effectiveness of devices.

Today, data related to cost-effectiveness assessments have been produced for the CGM systems.

The Diamond study that evaluated the societal cost-effectiveness of CGM in T1D patients with suboptimal glycemic control using MDI compared with SMBG reported that CGM is cost effective with improved glucose control and reductions in non-severe hypoglycemia [92].

The cost-effectiveness studies of SAP are inconclusive. Kamble et al., reported that despite the clear clinical superiority over MDI, SAP is not economically viable in the USA in patients with T1D [93]. However, a study conducted on T1D patients with suboptimal glycemic control or who experience frequent severe hypoglycemic events demonstrated that SAP compared to CSII has a favorable cost-effectiveness because it confers long-term clinical benefits [94].

The opinion regarding the economic aspect of SAP with LGS is also still controversial. A NICE document [95] reported that, despite being better than all the other systems (MDI, CSII, CGM, SAP) in reducing episodes of hypoglycemia in adults, SAP with LGS is not economically viable. However, a British study showed that in poorly controlled patients, SAP with LGS is cost-effective compared to CSII [96]. In addition, an Australian study demonstrated that SAP with LGS may be considered a cost-effective alternative to CSII with SMBG in unaware hypoglycemia patients with T1D [97]. Recently the cost-effectiveness of a hybrid closed-loop system has also been evaluated. An Australian study conducted on T1D patients reported that hybrid closed-loop therapy is likely to be cost-effective compared to MDI and SMBG [98] (Table 1).

The data published so far require further studies for expanding knowledge on the subject.

## 6. Emerging and Future Glucose Monitoring Systems

In current clinical practice glucose sensors and insulin infusion cannulas are inserted separately. However, single-port devices are being developed that combine glucose sensing and insulin infusion capabilities on the same platform in order to simplify device insertion and site management [21]. Studies conducted with the goal to evaluate single-port devices performance and its acceptability by patients showed a feasibility of insulin infusion and glucose sensing capabilities in a single device and accurate glucose readings during routine and home use. Moreover, it has been reported that the single-port device was acceptable to most patients and improved satisfaction and convenience [99,100].

In the last few years the progress related to CGM technologies focused on subcutaneous implantable electrochemical glucose sensors in order to optimize biocompatibility and glucose monitoring sensitivity, precision, accuracy, and durability [101].

The development of carbon nanotubes (CNTs) and graphene-based electrodes have been intensively studied, and they demonstrated higher sensitivity and faster response time than traditional electrodes [102].

Moreover, microgel as glucose sensing system has been developed; the peculiarity of this glucose sensor, and what differentiates it from other examples of traditional glucose sensors, is that it provides minimally invasive implantation and a fluorescent signal by transdermal transmission without any external links or electric power sources for CGM [103].

Recently, non-invasive glucose sensing systems have gained significant research interest due to the high sensitivity and better patient compliance, contrary to invasive ones. Major efforts have been devoted to developing entirely non-invasive wearable epidermal glucose sensors toward advanced glycemic control [104].

These emerging sensors can continuously detect glucose in skin interstitial fluid, tears, saliva, and sweat, and include epidermal temporary tattoos, adhesive sensor patches, wrist-worn electrochemically systems, contact lenses, and dental tattoos [105]. Moreover, a new method has been developed to measure the concentration of glucose in hair follicles that accurately reflects the plasma glucose measured by traditional glucose meters [106].

Despite their potential, these technologies are still being studied in order to refine their problems and limitations [104].

## 7. Conclusions

As reported in this review the CGM provides a method of collecting and using glucose data which requires a correct reading and interpretation by the diabetology team, in order to make therapeutic decisions as close as possible to the patient ’s metabolic needs. On the other side, glycemic data are available also for the patient, and he/she can use them to make appropriate therapeutic decisions. For this purpose, we proposed the concept of IGS, that includes the three CGM parameters described above, and that can be used by the patient to make therapeutic decisions. Currently, there are therapeutic intervention algorithms for specific devices that do not consider the patient’s IGS. Therefore, following the above, our purpose is to identify a method to calculate the value of IGS, to drive a specific therapeutic intervention by the patient based not only on insulin adjustment, but also on physical activity and diet. Accordingly, the patient will acquire an education that will allow him/her to interpret and manage his/her glycemic state.

In addition, data sharing between the patient, supported by family, caregivers, and cohabitants, and multidisciplinary diabetes team, has been described as a model of “circular flow”.

CGM studies highlight the safety and effectiveness of the device in different populations and reported an improvement on glycemic control and a reduction on hypoglycemic episodes, especially when combined with CSII, compared to SMBG. However, clinical outcomes related to CGM should also include the portability of the device and the level of technology provided, as they represent factors that can influence adherence to the use of the device and the patient’s quality of life. Studies aimed at defining patient’s characteristics that make him/her more compatible with specific devices, in terms of portability and level of technology, are needed, to reduce the number of dropouts among patients using CGM.

## Figures and Tables

**Table 1:** Cost-effectiveness of CGM, SAP, and hybrid closed-loop system.

References	Population	Technology	Cost-Effective
[92]	T1D with suboptimal glycemic control	CGM vs. SMBG	Yes
[93]	T1D	SAP vs. MDI	No
[94]	T1D with suboptimal glycemic control or with frequent severe hypoglycemic events	SAP vs. CSII+SMBG	Yes
[95]	T1D	SAP LGS vs. MDI, CSII, CGM, SAP	No
[96]	Poorly controlled T1D	SAP LGS vs. CSII	Yes
[97]	T1D with unaware hypoglycemia	SAP LGS vs. CSII+SMBG	Yes
[98]	T1D	Hybrid closed-loop vs. MDI+SMBG	Yes

CGM, continuous-glucose monitoring; CSII, continuous subcutaneous insulin infusion; LGS, low-glucose suspension; MDI, multiple daily injections; SAP, sensor-augmented pump; SMBG, Self-monitoring blood glucose; T1D, Type 1 diabetes.
